# Studies on Myxo-Angioma of the Skin

**Published:** 1882-11

**Authors:** C. Heitzmann


### STUDIES ON MYXO-ANGIOMA OF THE SKIN—CLINICAL AND MICROSCOPICAL.

Angioma is a tumor of the skin of frequent occurrence. It was formerly termed *teleangiectasis*, from the erroneous view that the blood vessels are simply dilated. At present we know that a new formation of blood vessels takes place in the growth. Such growths appear either as dark red spots on the skin, varying greatly in size, or as slight dark red elevations above the level of the skin, sometimes being even pediculated. Their characteristic feature is compressibility, *i. e.*, the blood can be forced out of them, and they can be made to disappear by pressure ; but as soon as the pressure ceases, the blood rushes back into the vessels, and the spot or tumor reappears. As a rule, they are not congenital, but they appear in early infancy, and remain stationary, or extend with advancing age. Exceptionally, however, angioma is congenital, and known in the vernacular as "fire mole."

The pathology of these tumors is known only in part. We distinguish three varieties : 1. Simple angioma ; 2. Lobular angioma ; 3. Cavernous angioma. Simple and lobular angioma may be composed of prevailing arterial, venous, or capillary blood vessels, and the color varies accordingly from a bright crimson to a dark purple. Cavernous angioma is invariably composed principally of veins.

Simple angioma exhibits the blood vessels uniformly distributed, and between them a delicate fibrous, or myxomatous connective tissue. The latter variety is the most frequent, consisting of smooth and even protrusions, sessile or pedunculated, of a jelly-like consistence, easily compressible and semi-translucent. The growth is situated in the outermost layers of the derma.

The lobular angioma consists of coils of blood vessels having a delicate fibrous connective tissue within the coils ; between the coils the connective tissue is in coarse bundles. These tumors reach a large size, exhibit a lobulated or nodular surface, and, as a rule, occupy the whole thickness of the derma.

Cavernous angioma is composed of sinuous venous plexuses, closely resembling the structure of the cavernous bodies of the penis, occupying the deeper layer of the derma and the subcutaneous tissue ; the latter variety is sometimes found in liver tissue and on mucous surfaces.

Angioma is a benign tumour, which in the majority of cases causes only disfigurement. Cavernous angioma is sometimes exceedingly painful. Any variety of angioma may, in exceptional cases, ulcerate spontaneously, and cause obliteration of the vessels and spontaneous cure. Hemorrhage is of no consequence in ulceration of simple or lobular angioma ; but fatal cases have occurred in ulcerating cavernous angioma.

The aim of therapeutical interference is the destruction of the blood vessels by means of excision, cauterization, vaccination, or the production of ulceration by irritating plasters. Small tumors of this kind are best removed by cauterization with fuming nitric acid, which is applied by means of a small hard-wood stick. If proper care is used, the resulting scar is trifling. Pediculated tumors are best removed with curved scissors, and subsequently touching the base with solution of perchloride of iron. Large tumors must be excised with the knife. Repeated attempts have been made to remove such tumors by electrolysis, and unquestionably favorable results have been obtained. It remains a question, however, whether the cures thus obtained are permanent, and not followed by a recurrence of the new formation.

Myxomatous tissue, one of the constituent elements of myxoangioma, appears in several forms ; as medullary tissue, as reticular tissue, as lymph or adenoid tissue, and as a reticular tissue filled with lymph corpuscles, as found in the thyroid body. In its normal development, myxomatous tissue appears in the earliest stages of embryonal life, and in the organs for the attachment of the embryo to the womb—the umbilical cord and placenta. In the fully developed organism, only the vitreous body and the lymph tissue are found as representatives of myxomatous tissue, appearing both in the form of lymph follicles and lymph ganglia, and of the erroneously so-called adenoid tissue, which is widely spread over the mucous layers, mainly those of the alimentary tract and of the uterus. The reticular variety, which holds a jelly-like basis substance in its meshes, is the most common form found in the benign growths called myxomata, and it is this variety which enters into the construction of the myxo-angioma. With this myxomatous tissue there is a new formation of blood vessels characterized by a large calibre, distinct endothelial walls, and a winding course. Very exceptionally pigment is formed in myxo-angioma, and this feature invariably indicates a tendency toward malignity, *i. e.*, toward the formation of melanotic sarcoma, or myeloma.—*C. Heitzmann, M. D., in Medical Chronicle.*

